# Patient safety is our business! Staff perspectives on implementing hospital falls prevention education

**DOI:** 10.1093/heapro/daae200

**Published:** 2025-01-17

**Authors:** Cheng Yen Loo, Steffanie Coulter, Carol Watson, Sharmila Vaz, Meg E Morris, Leon Flicker, Tammy Weselman, Anne-Marie Hill

**Affiliations:** School of Allied Health, University of Western Australia, 35 Stirling Highway, Perth, Western Australia, 6009 Australia; WA Centre for Health and Ageing, University of Western Australia, 48 Murray St Perth, Western Australia, 6000Australia; WA Centre for Health and Ageing, University of Western Australia, 48 Murray St Perth, Western Australia, 6000Australia; Royal Perth Bentley Group, East Metropolitan Health Service, 10 Murray Street, Perth, Western Australia, 6000 Australia; Royal Perth Bentley Group, East Metropolitan Health Service, 10 Murray Street, Perth, Western Australia, 6000 Australia; WA Centre for Health and Ageing, University of Western Australia, 48 Murray St Perth, Western Australia, 6000Australia; Ngangk Yira Institute for Change, Murdoch University, 90 South Street Murdoch Perth, 6150 Western Australia, Australia; Academic and Research Collaborative in Health (ARCH), Care Economy Research Institute (CERI), La Trobe University, Melbourne, Victoria, Australia; Victorian Rehabilitation Centre, Healthscope, 499 Springvale Road, Glen Waverley, Melbourne, 3150 Victoria, Australia; WA Centre for Health and Ageing, University of Western Australia, 48 Murray St Perth, Western Australia, 6000Australia; Royal Perth Bentley Group, East Metropolitan Health Service, 10 Murray Street, Perth, Western Australia, 6000 Australia; Geriatric Medicine, Medical School, University of Western Australia, 35 Stirling Highway, Perth, Western Australia, 6009, Australia; School of Allied Health, University of Western Australia, 35 Stirling Highway, Perth, Western Australia, 6009 Australia; WA Centre for Health and Ageing, University of Western Australia, 48 Murray St Perth, Western Australia, 6000Australia; School of Allied Health, University of Western Australia, 35 Stirling Highway, Perth, Western Australia, 6009 Australia; WA Centre for Health and Ageing, University of Western Australia, 48 Murray St Perth, Western Australia, 6000Australia

**Keywords:** socio-ecological framework, accidental falls, patient education, hospital, organizational change management, behavioural change, consumer

## Abstract

Providing patients with falls prevention education reduces falls in hospitals, yet there is limited research on what influences successful implementation at the staff, ward and hospital levels. We engaged hospital-based health professionals to identify multi-level barriers and enablers to patient falls education that could influence the implementation of a Safe Recovery program. Purposive sampling was used to recruit hospital staff (*n* = 40) for focus groups and one-on-one interviews. Data were analysed using content analysis and categorized using a socio-ecological framework to understand the micro, meso and macro level influences of hospital falls prevention. We identified five overarching themes, on the barriers and enablers to implementation of the Safe Recovery program. The enablers to falls prevention education included sharing the responsibility to implement the program, setting clear goals for staff, showing the impact of delivering the program, involving family to reinforce the messaging, using falls champions to upskill staff and making the resources (video and booklet) readily available to patients at all times. Barriers included insufficient time for staff to deliver falls prevention education, lack of falls prevention training for staff during their clinical training, absence of standardized protocols and clinical guidelines across hospital settings and insufficient hospital marketing to promote the program. Using a systems thinking approach, this study showed that implementation requires more advocacy and support for patient falls prevention across different tiers of the hospital system to integrate into usual care.

**Contribution to Health Promotion**

- We report barriers and enablers to implement new falls prevention programs in hospitals using a systems thinking approach.
- Despite being theoretically sound, many interventions do not produce real-world change because few are successfully implemented.
- Although some studies have reported on the barriers and enablers to implementing patient falls prevention programs, most have limited their analysis to the individual and ward level.
- Using a socio-ecological theoretical framework, this study reports on the barriers and enablers on an individual, ward and hospital level with the findings showing hospitals as complex systems with multiple tiers of influence.
- Harnessing these complex systems can inform the sustainability of health promotion programs to reduce falls in hospitals.

## BACKGROUND

Patient falls in hospitals are a serious and persistent concern, with worldwide recognition of this problem (Montero-Odasso *et al*., 2022; Morris *et al*., 2024). Falls rates in hospital range from 3 to 16 per 1000 patient bed days (Morris *et al*., 2024) and are higher in geriatric and rehabilitation wards (Hill *et al*., 2015). Approximately 30–50% of falls in hospital result in physical injuries ranging from bruises and lacerations to fractures and death (Morris *et al*., 2022, 2024). Experiencing a fall at hospital can cause psychological distress to the patient, their caregivers and staff (Oliver *et al.*, 2010; Morris and O’Riordan, 2017). Falls-related injuries are a significant burden on hospital resources and cost to the healthcare system because patients who fall in hospital have longer lengths of stay in hospital than patients who do not fall (Morello *et al*., 2015; Australian Commission on Safety and Quality in Healthcare, 2018). These consequences of in-hospital falls highlight the urgency to reduce falls to improve in-patient safety and lower the financial and resource burden on hospitals.

Hospitals falls prevention strategies can include the use of alarms and monitoring devices (e.g. bed alarms, chair alarms, sensors and wristbands), environmental safety practices (e.g. obstruction-free floors, rails, ramps), use of scored falls risk assessments (Morris *et al*., 2021) and medication reviews (Morris and O’Riordan, 2017; Turner *et al*., 2022). These methods show little effect in reducing falls (MacDonald, 2014; Schoen *et al.*, 2016; Cameron *et al*., 2018). In contrast, the delivery of patient and staff education about the risks and strategies to reduce falls has been found to be an effective means to reduce fall rates in older hospital patients (Hill *et al*., 2015; Hill, Francis-Coad, *et al*., 2016; Heng *et al*., 2020). Randomized controlled trials have also demonstrated the efficacy of falls prevention education for reducing patient falls (Haines *et al.*, 2004; Hill *et al*., 2009, 2015). The intervention used in these trials is the Safe Recovery program—an individualized patient education and goal-setting intervention in multimedia format delivered by training educators (Hill *et al*., 2009). The Safe Recovery program developed in Australia has proven to reduce falls rates by 40% and falls-related injuries (Haines *et al*., 2011; Hill *et al*., 2015; Francis-Coad *et al*., 2023). These trial findings aligned with the World Guidelines for Falls Prevention (Montero-Odasso *et al*., 2022) that recommend delivering tailored falls prevention education to effectively reduce falls by older patients in hospitals (Grade 1A evidence).

While falls prevention education is an effective strategy to reduce patient falls, the delivery of falls prevention education in hospitals is not systematically embedded as part of usual care. In fact, a recent systematic review of hospital clinical practice guidelines found that falls prevention education was not mentioned in all guidelines and many lacked clarity about how patient education should be implemented (McKercher *et al*., 2024). There is an emerging understanding that organizational and system learning in health care results from interactions of various factors, which operate at multiple levels within and beyond individual care delivery (Anderson *et al*., 2022). While hospitals are organizations responsible for the maintenance and recovery of the public’s health (Weisz and Haas, 2016), providing clinical care including falls prevention education is not the only criterion that informs decision-making. Questions of sustainability are central to hospital decision-making where human resource, economic and social cost to society form part of determining the longevity and sustainability of new interventions introduced into the hospital social ecology (Weisz and Haas, 2016).

The purpose of this study was to determine micro, meso and macro level barriers and enablers to the implementation of the Safe Recovery falls prevention program from the perspective of staff. We also aimed to identify areas for potential improvement of hospital falls prevention and methods to optimize the implementation of the Safe Recovery program into hospitals.

## METHODS

### Ethical considerations

Ethical approval was received from the Human Research Ethics Committee of Royal Perth Hospital (RGS No: 5775). All participants provided written informed consent to participate in the study.

### Study design

A qualitative descriptive approach (Doyle *et al.*, 2020) was used to examine the perception of health professionals implementing the Safe Recovery program in hospitals. Using a descriptive approach allows researchers to understand the subjective perspectives of implementing new programs, the diverse experiences of delivering falls prevention education, and how the findings address the objective of the study (Doyle *et al*., 2020). Using a descriptive study design is also commonplace in nursing studies to validate behavioural and system change.

### Theoretical framework

The study used a socio-ecological approach, which is a well-established theory-based framework that explores the multidimensional study effects of environmental and social factors that determine change (Kilanowski, 2017; Reyes *et al*., 2023). Taking a systems thinking approach (McNab *et al.*, 2020) to explore the factors that can influence the successful implementation of the Safe Recovery program helps to provide a multi-level understanding of barriers and enablers to program implementation. Different versions of the socio-ecological model have been applied in a broad range of research domains, including public health promotion (Rusoja *et al*., 2018), nursing (Chejor *et al.*, 2022) and organizational development (Bui and Galanou, 2022), and we adapted the socio-ecological model for the target of providing falls prevention education to hospital settings.

**Fig. 1:**
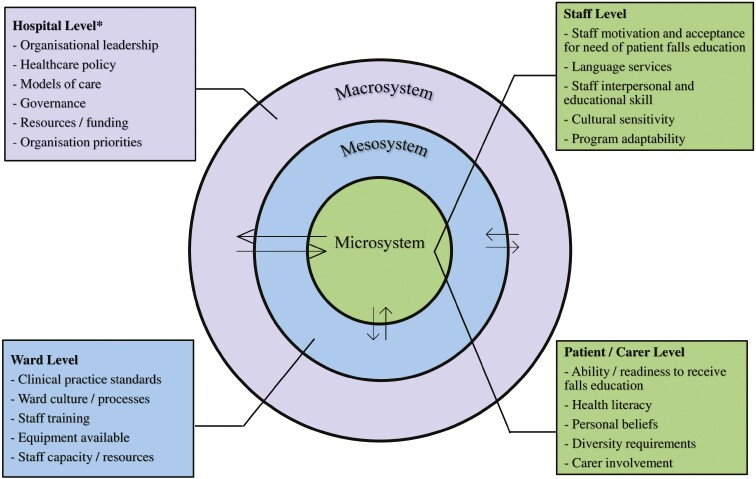
Hospital barriers and enablers to Safe Recovery program implementation.

Our model posited that factors that inform the successful implementation of the Safe Recovery program are influenced by the interaction of three nested and interrelated systems, being the microsystem (health professionals who provide care to hospital patients); the mesosystem (the interrelationship between health professionals and the ward environment) and the macrosystem (the hospital environment and broader health system where health professionals work). Responses outside the hospital setting were not delineated as a separate system and were instead included as part of the macrosystem to capture a holistic understanding of the interrelated influences that could inform and enable the planned implementation of the Safe Recovery program (Figure 1).

### Setting and sampling

Supported by allied health and nursing management, the research team (A.M.H., C.Y.L., T.W.) and a hospital staff member (S.C.) advertised the study in a 450-bed hospital in Western Australia using recruitment materials and word-of-mouth. Via purposive sampling (Campbell *et al*., 2020), health professionals with >6 months of experience working in medical wards that provided clinical care to older patients (+65 years) were recruited. Participants included physiotherapists (*n* = 18; 45%), registered nurses (*n* = 13; 32.5%), occupational therapists (*n* = 8; 20%) and a doctor (*n* = 1; 2.5%) (Supplementary File S1: Table S1). Two focus groups were hosted during hospital working hours and 20 individuals consented to being interviewed.

Focus groups and interviews were hosted in the hospital at a convenient time by researchers A.H.M., T.W. and S.C. using a guided questionnaire (Supplementary File S2: Table S2). The question guide contained four discussion facets (Supplementary File S3: Table S3) designed to elicit open dialogue between the participants about the implementation of the Safe Recovery program (Vaismoradi *et al.*, 2013; Gjessing *et al.*, 2023). The program was explained to the participants and a copy of the resources (video and booklet) was provided. The focus groups allowed the researchers to identify consensus between participants (Vaismoradi *et al*., 2013). The one-on-one interviews were held with the health professionals who were unable to attend the focus group.

### Data collection and analysis

The focus groups and interviews were audio-recorded and transcribed verbatim using an online transcription service. The transcripts were independently reviewed by A.M.H., C.Y.L. and S.C. alongside the original recordings to ensure accuracy. Data were analysed via content analysis, a qualitative descriptive approach (Kleinheksel *et al.*, 2020). This analysis approach is commonly used in nursing research and is well suited to the analysis of multi-faceted, important and sensitive phenomena (Vaismoradi *et al*., 2013). The transcripts were independently read by C.Y.L. and S.C. and short phrases significant to the study objective were assigned a code (Saunders *et al*., 2023). The codes were organized into meaningful groups that described the barriers and enablers to the implementation of the Safe Recovery program. Using investigative triangulation, the project team (A.H.M., C.Y.L., S.C. and M.M.) corroborated and refuted each other’s findings to achieve intercoder data agreement (Archibald, 2016). The coded statements were then grouped into five themes and nested under three primary categories: micro (individual), meso (team/ward) and macro (institution) enablers and barriers to implementation (Kilanowski, 2017). This study was conducted and reported following the COREQ guidelines for reporting qualitative research studies (Tong *et al.*, 2007).

## RESULTS

### Microsystem (individual) barriers and enablers to falls prevention education

#### Theme 1: Individual staff acceptance

Many participants reported that it was important to promote the benefits of delivering a patient falls education program to staff. They perceived that staff needed to be convinced that the benefits of providing falls prevention education outweighed the added responsibility of doing so. According to P19, ‘*there needs to be a way of selling it* [the Safe Recovery program], *especially for some professions to make their job more satisfying knowing that their patients won’t fall*’.

The participants expanded this rationale by explaining that often, staff are informed that a new initiative will be deployed without a substantive explanation for why and how it could benefit the patients. ‘*A lot of the time, we get told what is happening and what to do without any background about who’s been consulted and why it is happening*’ [P3]. A suggestion for creating program buy-in was to ‘*publish some of the KPI’s and patient fall statistics on a dashboard*’ [P8] to show staff measurable evidence of how their efforts could directly improve patient safety.

To encourage hospital staff to embrace the Safe Recovery program, some participants advised that it was important to understand what attracted each type of hospital employee to their profession and what aspect of patient care they are most invested in. The reason for garnering this information was to determine the best way to communicate the benefits of the Safe Recovery program to different types of health professionals, ‘*You’ve got to figure out how to talk to people so they see the point of it…what attracted an ED nurse to the job is different from a nurse working in the gerontology ward*’ [P19].

#### Sub-theme 1(a): Staff responsibility and workload balance

There was concern from a small number of staff about the time required to implement the Safe Recovery program and where falls education was ranked in the list of patient care priorities, ‘*if push came to shove between seeing a new stroke patient and sitting them out of bed versus giving education, it’s going to become lower priority unless it was one person’s specific duty*’ [P36]. However, this sentiment was not shared by everyone as many participants said delivering falls prevention education for patient safety was important, ‘*falls education is usually low on the list of priorities, but it’s extremely important, particularly when patients are at their most vulnerable’* [P4].

Sharing the responsibility was deemed necessary to reduce placing too much responsibility on individual health professionals when they are be preoccupied with multiple tasks, ‘*the moment you say that this is physio or the occupational therapists’ job, it places the onus solely on them… if they are too busy to fulfill the task, others might back away and say that it’s not my problem*’ [P3]. However, there was an overwhelming consensus from most participants that physiotherapists and occupational therapists were the most suitable health professional to implement the Safe Recovery program due to their training and knowledge of patient mobility, ‘*It would have to be a physio or an OT to introduce the program…with physio assistants providing support’* [P6].

#### Theme 2: Program adaptability for diverse patient needs

Some participants identified that a barrier to program delivery was accessibility of the program for patients with mild cognitive impairments, as the program is recommended to be delivered to patients with no or mild cognitive impairment. Others recommended implementing the program during times when patients with cognitive impairment are most alert to overcome this barrier, ‘*in the afternoon, dementia patients are probably not going to absorb the information verses first thing in the morning when they’re awake and have had something to eat*’ [P6]. It was also suggested to involve family and informal carers to assist in reinforcing the education, ‘*For the cognitively impaired patient, I think family’s quite important and makes a huge difference for their own understanding’* [P6].

Others identified a need to make the Safe Recovery program accessible for patients with limited English language skills and those who come from non-Anglo Australian cultural backgrounds. According to P34, ‘*we get a lot of patients that speak another language* [other than English] *and this could be a barrier to delivering the program*’. In a similar sentiment, P35 said, ‘*a lot of the patients we get here are Indigenous and I think we would need to think about some other way to better communicate the information for them*’.

### Mesosystem (interpersonal team/ward) barriers and enablers to program implementation

Participants offered a range of suggestions about enablers and barriers at ward level that could impact on implantation of the Safe Recovery program, and the following themes were identified.

#### Theme 3: Ward procedure to support staff

Some participants emphasized the importance of having falls champions in hospital wards to upskill and communicate the importance of the program to health professionals. According to P7, ‘*something to consider would be having a Safe Recovery staff member on the ward to implement the program in the early stages and to share the learnings with other ward staff*’ [P7]. Another participant stated, ‘*If you roll out the program one ward at a time, you could train up certain people and use them as champions*’ [P1].

Many participants also emphasized the need to set clear expectations, roles and responsibilities for ward staff to encourage individual staff accountability, ‘*I do think for it* [Safe Recovery program] *to be effective, it needs strong recommendations from management that new expectations have been created and all patients who meet the criteria is to have it done within specific timeframes*’ [P19]. Participants also recommended integrating the Safe Recovery program delivery as a key performance indicator for relevant wards by setting completion targets coupled with training to educate staff about the importance of delivering the program. According to P7, ‘*If we have clear baseline targets or KPIs to say this needs to be delivered to everyone in this cohort or with certain risk factors*’.

#### Sub-theme 3(a): Staff training to bridge knowledge gaps

A recurring theme identified was that health professionals had gaps in their knowledge of falls prevention education due to limited education in falls prevention provided during their clinical training. According to P39, ‘*I don’t think we do falls education enough within medicine… reflecting on my training, falls education only came to the forefront when I started advanced training in geriatrics*’.

Participants identified the need to develop consistent procedures and protocols for their wards across all health service settings. One participant stated, ‘there is *no formal content, pamphlets or booklets are available…it’s all just verbal education*’ [P28]. Furthermore, ‘*Having worked in many hospitals, everybody’s got their own way of doing things and when they move to a different hospital, its different again*’ [P28]. Many participants expressed the need to standardize falls prevention protocols in all hospital settings, ‘*at my previous workplace, we had a post falls protocol that was 9 steps. We also had laminated cards to follow which we don’t use here* (current workplace)’ [P33].

A few participants elaborated on their perspectives by advising that it could be helpful for the government department of health to focus on establishing an overarching consistent falls prevention policy for all public health services. According to P28, ‘*if it was a WA* [Western Australian] *government-wide or Perth-wide initiative, then I would feel like everybody would be on the same page about the* [falls prevention] *program*’.

#### Theme 4: Hospital procedural considerations

Most participants said that the Safe Recovery program booklet and video were useful tools that delivered clear and comprehensive messaging on how to stay safe at hospital. However, there were suggestions for health professionals to have additional materials and procedures to facilitate implementation. One suggestion was to *‘Add the program to the comprehensive care plan so that implementation forms part of usual care’* [P4]. Another suggestion was to place a marker on patient-integrated notes to highlight which patients had received the Safe Recovery program, ‘*Maybe stickers could be made available to put in the case files at the nursing coordinator’s desk or to have a notebook that lets you screen which patient has received it*’ [P7].

While there were no concerns identified regarding delivering the Safe Recovery program individually to patients, delivering the program in groups was suggested to increase the number of patients that could receive falls prevention education in one sitting, ‘*I feel like if you delivered it in a group setting, it would be more time efficient*’ [P1]. Similarly, P3 said, ‘*Showing the video in a group session would help us hit the number of people (KPI targets) …we can then follow up the booklet with patient engagement afterward*’.

#### Sub-theme 4(a): Supportive educational resources

Many participants discussed how the video could support the implementation of the Safe Recovery program in their ward. One possibility was to encourage patients to watch the video on a DVD player while wearing headphones, ‘*I think using a DVD player and headphones is a great way of delivering it…it will be one-on-one, and the patient can ask questions at the same time*’ [P11]. However, some participants said that using a DVD player was impractical, ‘*it would be a hassle pushing a trolley with a TV and DVD player around the ward*’ [P33]. An alternative option was to have ‘*an iPad or similar electronic device that could be easily passed around the patients*’ [P28]. Several participants suggested that ‘*it’d be good to have it (the video) on the TV’s, sort of as a background channel on repeat… kinda like the plane videos*’ [P7].

Complementing the Safe Recovery program video, several participants advised the booklet was a useful tool. It provided information about the falls prevention education which was particularly useful when patients first arrived at the ward. It could also be used to reinforce fall prevention messaging during their stay. According to P2, ‘*having the booklet when they (patients) first come to the ward so they know the main points …works better for our patients*’. This sentiment was reinforced by P6, ‘*the booklet is easy to read. It’s good that it has pictures, it’s not too descriptive and the important parts are highlighted*’.

Some participants suggested that the booklet could be made accessible for family and informal carers when they visit, ‘*the booklet is really important…to relook over if they (family and friends) come together is a good strategy*’ [P34]. Making sure the booklet was consistently available to patients during their stay at hospital was also highlighted, particularly if the patient was unable to watch the Safe Recovery program video or when they felt bored, ‘*if someone is less attentive, they might be able to flick through the book…and for patients who are bored, they may look at the booklet in greater detail*’ [P4].

### Macrosystem (institutional) barriers and enablers

Suggestions were offered on the type of support health professionals may need on an institutional/organizational level to enable implementation of the Safe Recovery program.

#### Theme 5: Hospital leadership and advocacy

A recurring theme was the need for sufficient organizational support to improve awareness and knowledge about the Safe Recovery program among hospital staff. There was strong consensus that the hospital needed to provide resources to market and promote the program not just to clinical staff but to all hospital employees, ‘*I believe from a hospital level, there needs to be the marketing and messaging and you need to have those people who are going to champion the cause to follow through and figure out what are the barriers*’ [P3]. Similarly, P34 said, ‘*it needs to be advertised so everybody knows about its hospital-wide…perhaps the program could be linked to the hub page or sent out on global* [email]’.

Some participants reported that the Safe Recovery program needed to be elevated to take equal status with other hospital priorities to enable implementation, ‘*if it’s going to be successful, there needs to be a push to make it a priority alongside the other hospital strategies*’ [P3]. In addition, many participants also expressed the need for organizational accountability to ensure adequate staff, time and technological resources be made available to enable the implementation of the program. This recommendation was in response to an identified barrier to program implementation, being staff and time constraints, ‘*I think that time is always going to be a barrier…for nurses and allied health staff, they’re always busy*’ [P28].

## DISCUSSION

Overall, health professionals agreed that effective implementation of the Safe Recovery falls prevention program is influenced by barriers and enablers at micro (individual), meso (ward) and macro (institutional) level. The micro-level influences comprise individual knowledge, attitudes, beliefs and perceptions of interventions that can influence behaviour (Salihu *et al.*, 2015). In the context of integrating the Safe Recovery falls prevention program as part of usual care, many participants reported that showing clear changes in falls rates over time would motivate health professionals to prioritize delivery. This reflects global studies showing the benefits of goal-setting and incentive-based behavioural change techniques to reshape the beliefs of individuals (Vlaev *et al.*, 2019; Epton and Armitage, 2020). Many participants expressed the need to share the implementation responsibilities amongst all hospital staff, distribute the workload and designate falls champions to assist ward staff, in agreement with comparable research from other teams regarding falls prevention education (Heng *et al*., 2022; Shaw *et al.*, 2023).

The diversity of patients admitted to hospitals in our study reflects the cultural and linguistic diversity of our hospital catchment. The need to adapt the Safe Recovery program for patients with diverse cognitive, cultural and linguistic needs was raised by many participants. This aligns with the reports of prior studies showing that conveying information in a manner and language that is easily understood is vital to improving health literacy (Chu *et al.*, 2022; Kanengoni-Nyatara *et al*., 2024).

In this investigation, extant influences that interacted with individual health professionals to inform the successful implementation of the Safe Recovery program were categorized as meso-level influences. Many participants advised that introducing a systematic approach to deliver the program was important such as integrating this falls prevention program into patient comprehensive care plans. A concern raised by some participants was the inconsistency in health professionals’ falls knowledge. While all Australian hospitals are obligated to abide by the National Safety and Quality Health Standards (2021) that contain provisions to minimize patient harm (including falls); how those standards are met is managed at the site-specific level. Secondly, the extent health professionals are familiar with the national protocols varies according to their clinical background, years of experience managing falls incidents in hospital and exposure to the policies.

Emphasis was placed upon the state health sector and government to institutionalize training for clinical staff about falls prevention and the need to standardize falls prevention procedures across all health institutions. The outcome of doing so would initiate behavioural change throughout the hospital system and reduce patient fall risks. A review of existing healthcare policy may be needed to enforce systematic change on a state and national level. These findings are supported by the UK public health guidelines (Office for Health Improvement and Disparities, 2022) for falls prevention, which provide a systematic series of falls information and recommendations for all health professionals to address falls prevention programs.

Participants noted that while the Safe Recovery program’s booklet and video were useful, additional procedural resources were needed to ensure systematic delivery. A key sub-theme was the importance of using electronic equipment on the wards to support staff to implement the Safe Recovery falls prevention program. Another recent study that examined the role of digital communication tools in health care found that digital devices can help empower patients and foster better health outcomes for both patients and staff (Fitzpatrick, 2023). This finding is also supported from feedback in another recent study on the revised Safe Recovery program where many participants found the video to be interesting and easy to understand (Francis-Coad *et al*., 2023).

Similarly, the use of the Safe Recovery program booklet was deemed to be an effective tool for introducing the key messages to patients upon first arrival to the hospital ward and as a resource to reinforce the learnings after viewing the Safe Recovery video. This finding is supported by the study that reported on the revised version of the Safe Recovery program where staff and patients alike noted that the instructions were easy to follow and a useful tool to reinforce the key messaging (Francis-Coad *et al*., 2023).

Macro (organizational) level factors that informed the successful implementation of the Safe Recovery program were also discussed amongst health professionals. Many advised that messaging about the program required support at all levels of the hospital rather than single wards. The hospital had a role in promoting the program to all health professional employees. Care workforce redesign has been shown to facilitate this through diversifying the workforce to support widespread implementation (Morris *et al*., 2024) On a leadership front (Kumar, 2022), elevating the Safe Recovery program as part of core hospital strategies could facilitate financial investment to support the implementation of the Safe Recovery program at the ward level. To achieve this outcome, more emphasis may be warranted on how the Safe Recovery program can contribute towards the economic, social and ecological sustainability of hospitals (Weisz and Haas, 2016).

## STRENGTHS AND LIMITATIONS

There were several limitations of this study. Participants were recruited from one Australian public hospital which restricted the generalizability of findings to the public sector in the Oceania region. While purposive sampling ensured that health professionals with different levels of clinical expertise contributed, each ward and hospital setting has a different work culture which can influence participant responses (Hill, Waldron, *et al*., 2016; Heng *et al*., 2021). Recruitment did not involve engaging hospital executives and administrators or hospital staff who could also play a role in falls prevention. One of the strengths of the study was that data collection was rigorously audited and reviewed by three independent researchers to validate the themes to ensure visibility and transparency of the research design and findings (Creswell, 2009; Liamputtong, 2020). We also conducted the investigation according to the COREQ guidelines to promote explicit and comprehensive reporting (Buus and Perron, 2020).

## CONCLUSION

Health professionals reported that delivering falls prevention education is important to reduce hospital falls, limit patient hospital admission days and minimize return hospital admissions. A collaborative multi-level approach was recommended to facilitate the successful implementation of the Safe Recovery falls prevention program. There is a need to take a whole-of-organizational approach to identify the barriers and enablers to falls education implementation.

## Supplementary Material

daae200_suppl_Supplementary_Files_1

daae200_suppl_Supplementary_Files_2

daae200_suppl_Supplementary_Files_3

## Data Availability

The data underlying this article are available in the article and in its online supplementary material.
