# Supplementary material for: Patient safety is our business! Staff perspectives on implementing hospital falls prevention education
Source: Health Promot Int. 2025 Jan 17;40(1):daae200. doi: 10.1093/heapro/daae200 (PMC11739717; doi:10.1093/heapro/daae200)
Supplement: daae200_suppl_Supplementary_Files_1 [file daae200_suppl_supplementary_files_1.docx]

**Table 1: Health professional Participant demographics**

| **Participant characteristics** | **Participants (n = 40)** |
| --- | --- |
| Age range |  |
| 20-34 | 27 (67.5%) |
| 35-44 | 6 (15%) |
| 45-54 | 3 (7.5%) |
| 65+ | 2 (5%) |
| Prefer not to say | 1 (2.5%) |
| Gender |  |
| Female | 37 (92.5%) |
| Male | 3 (7.5%) |
| Profession |  |
| Physiotherapist | 18 (45%) |
| Occupational Therapist | 8 (20%) |
| Nurse | 13 (32.5) |
| Doctor | 1 (2.5%) |
| Education level |  |
| Bachelor’s degree | 30 (75%) |
| Graduate certificate/diploma | 3 (7.5%) |
| Post-graduate qualification | 6 (15%) |
| Other | 1 (2.5%) |
| Primary language |  |
| English | 37 (92.5%) |
| Other | 3 (7.5%) |
| Employment status |  |
| Full-time | 32 (80%) |
| Part-time | 8 (20%) |
| Primary Role |  |
| Direct patient care | 39 (97.5%) |
| Management | 1 (2.5%) |
| Work Area |  |
| Medical | 22 (55%) |
| Surgical | 4 (10%) |
| Gerontology | 7 (17.5%) |
| Emergency | 7 (17.5%) |
| Years in profession |  |
| 0-2 years | 3 (7.5%) |
| 3-5 years | 13 (32.5%) |
| 6-10 years | 11 (27.5%) |
| >11 years | 13 (32.5%) |
| Received falls education last 12 months |  |
| Yes | 14 (35%) |
| No | 26 (65%) |
