# Supplementary material for: Patient safety is our business! Staff perspectives on implementing hospital falls prevention education
Source: Health Promot Int. 2025 Jan 17;40(1):daae200. doi: 10.1093/heapro/daae200 (PMC11739717; doi:10.1093/heapro/daae200)
Supplement: daae200_suppl_Supplementary_Files_2 [file daae200_suppl_supplementary_files_2.docx]

**Table 2: Data collection method and staff recruitment**

| **Data Collection Method** | **Profession*** | **Number of Participants** |
| --- | --- | --- |
| Interview | PT, OT, CN, Dr | Participants (n = 19) |
| Focus Group 1 (12/07/2023) | RN, CN, SDN, NUM | Participants (n = 10) |
| Focus Group 2 (13/07/2023) | OT, PT | Participants (n = 10) |
| Interview and Focus group 1 | CN | Participant (n = 1) |
| Total |  | Participants (n = 40) |

*Physiotherapists (PT); Occupational therapists (OT); Clinical nurse (CN); Doctor (Dr); Registered nurse (RN); Staff development nurse (SDN); and Nurse unit manager (NUM).
