# Supplementary material for: Patient safety is our business! Staff perspectives on implementing hospital falls prevention education
Source: Health Promot Int. 2025 Jan 17;40(1):daae200. doi: 10.1093/heapro/daae200 (PMC11739717; doi:10.1093/heapro/daae200)
Supplement: daae200_suppl_Supplementary_Files_3 [file daae200_suppl_supplementary_files_3.docx]

**Table 3: Steps involved in the focus group and interviews.**

| Discussion 1 | Discuss your experiences with fall prevention education in hospitals |
| --- | --- |
| Discussion 2 | Exploring how clinicians identify patients at risk of falls and strategies to direct fall prevention education to these patients |
| Discussion 3 | Viewing the Safe Recovery (SR) program's resources and reflecting on the material (video + workbook)  Participants will be shown a newly revised patient-tailored, evidence-based fall prevention education resource called the Safe Recovery Program  Participants to share their views about the resource and the barriers and enablers to using it in WA hospitals, including how they think older people with and without common problems identified in Step 2 (cognitive impairment, medically unwell) would respond/ enact messages |
| Discussion 4 | Brainstorm how and where fall prevention messages might be delivered to older people during or before hospital admission and ways to support nurses and health professionals in delivering fall prevention education.  Commonly based barriers and enablers identified in previous systematic reviews and realist evaluations of SRP to be used to probe |
| Closing discussion | Group member checking and closing the focus group |

| **Interview Question Guide for Focus Group and 1:1 Interview** |
| --- |
| Step 1: Your experiences with fall prevention education in hospitals |
| Step 2: Exploring how clinicians identify patients at risk of falls and strategies to direct fall prevention education to these patients |
| 1. How do you identify patients at risk of falls? |
| 1. How could fall prevention education messages be directed to patients at risk of falls? |
| 1. Who do you think should be responsible for delivering fall prevention education to hospital patients? |
| Step 3: Viewing the Safe Recovery Program's resources and reflecting on the material (video + workbook) |
| Step 4: Brainstorm how and where fall prevention messages might be delivered to older people during or before hospital admission and ways to support clinical staff (nurses, allied health prof and doctors) in delivering fall prevention education |
| 1. Patient-level barriers and enablers |
| 1. Intervention-staff level barriers and enablers |
| 1. Intervention-level barriers and enablers |
| 1. System level barriers |
